# Decreased nesting behavior, selective increases in locomotor activity in a novel environment, and paradoxically increased open arm exploration in *Neurogranin* knockout mice

**DOI:** 10.1002/npr2.12150

**Published:** 2020-12-03

**Authors:** Ryuichi Nakajima, Satoko Hattori, Teppei Funasaka, Freesia L. Huang, Tsuyoshi Miyakawa

**Affiliations:** ^1^ Division of Systems Medical Science Institute for Comprehensive Medical Science Fujita Health University Toyoake Japan; ^2^ Department of Medicine University of the Ryukyus Okinawa Japan; ^3^ Program of Developmental Neurobiology NICHD NIH Bethesda MD USA

**Keywords:** ADHD, Alzheimer's disease, animal model, neurogranin, schizophrenia

## Abstract

**Aims:**

Neurogranin (NRGN) is a postsynaptic protein kinase substrate that binds calmodulin in the absence of calcium. Recent studies suggest that NRGN is involved in neuropsychiatric disorders, including schizophrenia, ADHD, and Alzheimer's disease. Previous behavioral studies of *Nrgn* knockout (*Nrgn* KO) mice identified hyperactivity, deficits in spatial learning, impaired sociability, and decreased prepulse inhibition, which suggest that these mice recapitulate some symptoms of neuropsychiatric disorders. To further validate *Nrgn* KO mice as a model of neuropsychiatric disorders, we assessed multiple domains of behavioral phenotypes in *Nrgn* KO mice using a comprehensive behavioral test battery including tests of homecage locomotor activity and nesting behavior.

**Methods:**

Adult *Nrgn* KO mice (28‐54 weeks old) were subjected to a battery of comprehensive behavioral tests, which examined general health, nesting behavior, neurological characteristics, motor function, pain sensitivity, locomotor activity, anxiety‐like behavior, social behavior, sensorimotor gating, depression‐like behavior, and working memory.

**Results:**

The *Nrgn* KO mice displayed a pronounced decrease in nesting behavior, impaired motor function, and elevated pain sensitivity. While the *Nrgn* KO mice showed increased locomotor activity in the open field test, these mice did not show hyperactivity in a familiar environment as measured in the homecage locomotor activity test. The *Nrgn* KO mice exhibited a decreased number of transitions in the light‐dark transition test and decreased stay time in the center of the open field test, which is consistent with previous reports of increased anxiety‐like behavior. Interestingly, however, these mice stayed on open arms significantly longer than wild‐type mice in the elevated plus maze. Consistent with previous studies, the mutant mice exhibited decreased prepulse inhibition, impaired working memory, and decreased sociability.

**Conclusions:**

In the current study, we identified behavioral phenotypes of *Nrgn* KO mice that mimic some of the typical symptoms of neuropsychiatric diseases, including impaired executive function, motor dysfunction, and altered anxiety. Most behavioral phenotypes that had been previously identified, such as hyperlocomotor activity, impaired sociability, tendency for working memory deficiency, and altered sensorimotor gating, were reproduced in the present study. Collectively, the behavioral phenotypes of *Nrgn* KO mice detected in the present study indicate that *Nrgn* KO mice are a valuable animal model that recapitulates a variety of symptoms of neuropsychiatric disorders, such as schizophrenia, ADHD, and Alzheimer's disease.

## Abstract 2

We found that Neurogranin knockout mice exhibit decreased nesting behavior, selective increases in locomotor activity in a novel environment, and paradoxically increased open arm exploration. Considering the behavioral phenotypes that had been previously identified, we propose that Neurogranin KO mice are a valuable animal model that recapitulates a variety of symptoms of neuropsychiatric disorders, such as schizophrenia, ADHD, and Alzheimer's disease.

## Graphical abstract

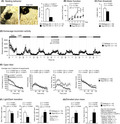

1

Neurogranin (NRGN) is a neuron‐specific protein that regulates calmodulin availability. Increases in postsynaptic calcium result in the release of calmodulin from neurogranin and participates in the protein kinase C signaling pathway.^1^, ^2^ A genome‐wide association study identified a significant association with schizophrenia at a locus near the *NRGN* gene in European populations.^3^ Children with Jacobsen syndrome, which involves attention deficit hyperactivity disorder (ADHD), have a deletion in the *NRGN* gene.^4^, ^5^ In patients with Alzheimer's disease (AD), NRGN protein levels are decreased in the brain tissue^6^, ^7^ and increased in the cerebrospinal fluid,^8^, ^9^ compared with healthy controls. Elevated NRGN peptide levels in the cerebrospinal fluid, which may reflect decreased NRGN protein levels in the brain,^10^ have also been reported in patients with mild cognitive impairment (MCI).^11^, ^12^, ^13^, ^14^ These findings imply that NRGN might be involved in the pathophysiology and pathogenesis of various neuropsychiatric disorders. Since the generation of *Nrgn* knockout (*Nrgn* KO) mice,^1^ several behavioral studies have been carried out on these mice, and hyperactivity,^15^ deficits in spatial learning,^16^, ^17^ impaired sociability,^15^ and decreased prepulse inhibition^18^ were identified, suggesting that these mice recapitulate some symptoms of neuropsychiatric disorders. To further validate *Nrgn* KO mice as a model of certain neuropsychiatric diseases, we assessed various behavioral domains in aged *Nrgn* KO mice using a comprehensive behavioral test battery^19^, ^20^, ^21^ (summary of the results and ages of the mice are available in the supplementary table).

We found that *Nrgn* KO mice exhibited a clear decrease in nesting behavior compared with that in wild‐type mice (Figure 1A; male, *P* < 0.0001; female, *P* = 0.0006), which may be analogous to the impaired executive function^22^, ^23^ seen in patients with schizophrenia,^24^, ^25^ ADHD,^26^, ^27^ and AD.^28^ In the rotarod test, the mutant mice exhibited a shorter latency to fall than that of the control mice (Figure 1B; *P* = 0.0031), which may be analogous to the motor dysfunctions in schizophrenia, ADHD, and AD.^25^, ^29^, ^30^ The *Nrgn* KO mice showed a slight but significant decrease in the latency of the paw response in the hot plate test (Figure 1C; *P* = 0.023).

**FIGURE 1:**
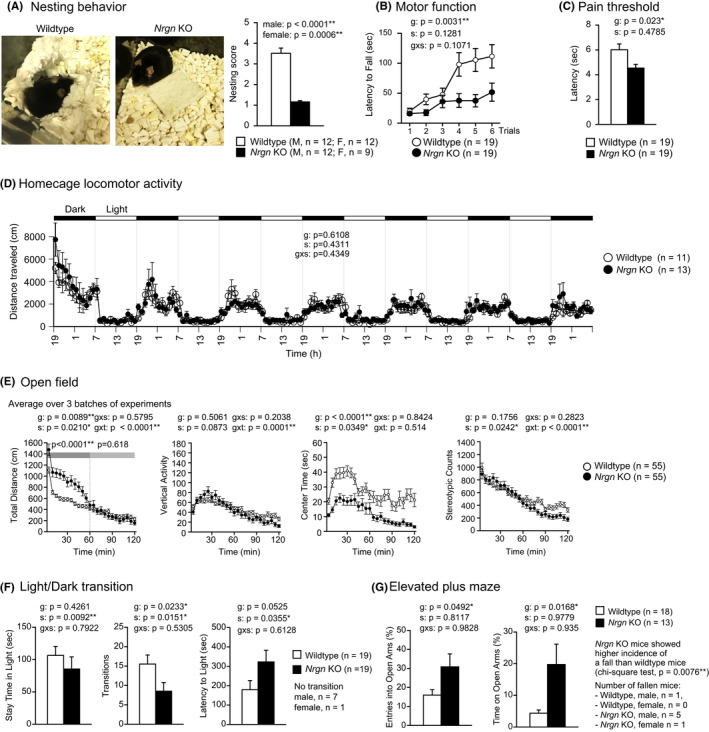
Decreased nesting behavior, selective increase of locomotor activity in a novel environment, and paradoxically increased open arm exploration in *Nrgn* KO mice (A) Nesting behavior. (B) Motor function. (C) Latency of the first fore or hind paw response in the hot plate test. (D) Locomotor activity in homecage. (E) Locomotor activity, vertical activity, time stayed in the central area, and number of stereotypic behaviors in the open field test. (F) Time spent in the light compartment, number of light‐dark transitions, and latency to enter the light compartment in the light‐dark transition test. (G) Percentage entries into open arms of the total number of entries to all arms and percentage of time on open arms of the total duration of the experiment in the elevated plus maze test. Data represent the mean ± SEM. ANOVA (in A, C, F, and G) or repeated measures ANOVA (in B, D, E) were used for the statistical analysis. g: genotype effect; s: sex effect; g × s: genotype and sex interaction; g × t: genotype and time interaction. For data where a significant sex effect was observed (body weight, body temperature; indexes in light/dark transition test, T‐maze test, and open field test), the male and female data are shown separately in the supplementary figures. Significant interactions between genotype and sex effects, which suggest the sex dependence of the expression of the genotype effect, were not detected in any of the tests (see supplementary table for the summary of all the results) [Colour figure can be viewed at wileyonlinelibrary.com]

In the open field test, mutants were significantly more active than controls in the first 60 minutes of the test (Figure 1E; whole period, genotype effect, *P* = 0.0089; the first 60 minutes, *P* < 0.0001; the last 60 minutes, *P* = 0.618; time × genotype interaction, *P* < 0.0001), suggesting that the expression of hyperlocomotor activity in *Nrgn* KO mice was limited to novel environments. This finding is further supported by the results from the homecage locomotor activity test, which did not detect a significant genotype effect on distance traveled (Figure 1D; *P* = 0.6108), indicating that the hyperactivity of *Nrgn* KO mice disappeared in a familiar environment. Concordantly, the results from the open field test and homecage locomotor activity test indicate that *Nrgn* KO mice show increased locomotor activity in response to a novel but not to a familiar environment. Increased locomotor activity is also a common characteristic seen in other schizophrenia^23^ and AD^31^ mouse models.

In the open field test, the *Nrgn* KO mice spent significantly less time in the center of the field during the 2‐hours session than the control mice (Figure 1E; *P* < 0.0001), which is generally interpreted as increased anxiety‐like behavior. In the light‐dark transition test, *Nrgn* KO mice tended to stay in the light compartment for less time than the control mice, but the difference was not statistically significant (Figure 1F, left panel; *P* = 0.4261). In the same test, *Nrgn* KO mice also exhibited a decreased number of transitions between light‐dark compartments (Figure 1F, middle panel; *P* = 0.0233) and a tendency of increased latency to enter the light chamber (Figure 1F, right panel; *P* = 0.0525), suggesting increased anxiety‐like behavior in the mutant mice, which has also been reported previously.^16^ In contrast, in the elevated plus maze, *Nrgn KO* mice showed significantly increased entries into open arms and time on open arms (Figure 1G; *P* = 0.0492 and *P* = 0.0168, respectively). The paradoxical changes in behavioral measures for anxiety‐like behavior in *Nrgn* KO mice may be attributed to the increased reactivity to novelty that we found in the present study, panic‐like behavior,^23^ or elevated impulsivity.^32^ The blood corticosterone concentration in the *Nrgn* KO mice after the elevated plus maze was not significantly different from that in the wild‐type mice (Figure S2C, *P* = 0.5517). The *Nrgn* KO mice also showed a significantly higher incidence of falling from the maze than the control mice (Figure 1G; *P* = 0.0076; chi‐square test). All of the *Nrgn* KO mice that fell stepped off the open arms backwards rather than forwards and clung to the arms to prevent the fall, suggesting that the increased incidence of falls in mutant mice is likely accidental and due to impaired motor function, as seen in the rotarod test. These behavioral phenotypes may recapitulate the altered anxiety states seen in patients with neuropsychiatric disorders.^33^, ^34^

We also reproduced behavioral phenotypes in the mutant mice that have been reported (available as supplementary materials). The *Nrgn* KO mice exhibited decreased prepulse inhibition^18^ (Figure S5; prepulse inhibition [%]; *P* = 0.0315), decreased immobility^15^ in the Porsolt forced swim test (Figure S5; immobility [%] on day 2; *P* = 0.067), and a tendency of decreased sociability and social preference in male mice^15^ (Figure S4, F, and G; time spent around a stranger cage [ratio]; *P* = 0.2069 and *P* = 0.3295, respectively). Female mutant mice showed a significantly decreased ratio of time spent around stranger cage both in sociability and social preference tests, while sexual attraction of the male stranger mice might have confounded their social behaviors (Figure S4, H and I; time spent around a stranger cage [ratio]; *P* = 0.0054 and *P* = 0.0145, respectively). Consistent with the tendency of impaired working memory in the radial‐arm maze reported previously,^17^ in the present study, *Nrgn* KO mice showed a statistically significant decrease in correct responses (Figure S6; correct responses [%]; *P* = 0.0062) as measured by the T‐maze spontaneous alternation test.

Overall, *Nrgn* KO mice recapitulate a variety of the typical symptoms of schizophrenia, ADHD, and AD, including impaired executive functions, motor dysfunction, increased activity in response to novelty, and altered anxiety levels, which we found in the present study. We also reproduced most of the behavioral phenotypes that were previously reported. Until recently, *Nrgn* KO mice have been suggested to be an animal model of schizophrenia and Jacobsen's syndrome with ADHD symptoms,^15^ as these mice show hyperactivity,^15^ altered anxiety‐like behavior,^16^ decreased sociability,^15^ impaired reference memory,^15^, ^16^, ^17^ and impaired sensorimotor gating.^18^ Behavioral phenotypes of commonly used AD model mice such as decreased nesting behavior,^31^, ^35^, ^36^, ^37^ hyperactivity,^31^, ^37^, ^38^, ^39^, ^40^, ^41^ impaired sociability,^35^ impaired working/reference memory,^31^, ^35^, ^38^, ^39^, ^42^, ^43^, ^44^, ^45^ and abnormal sensorimotor gating^46^, ^47^ overlap with those of *Nrgn* KO mice, which suggests the potential of *Nrgn* KO as a model of AD. Considering this, a decrease in NRGN in the brains of AD model mice^48^ and AD patients^6^, ^7^ may potentially explain some of the phenotypes or symptoms, respectively. Taken together, the behavioral phenotypes of *Nrgn* KO mice indicate that *Nrgn* KO mice might be a valuable animal model for further investigation of the pathophysiology and pathogenesis of neuropsychiatric disorders, including schizophrenia, ADHD, and AD.

## CONFLICT OF INTEREST

The authors have no conflicts of interest with regard to the present article.

## AUTHOR CONTRIBUTIONS

RN wrote the manuscript. SH, FLH, and TM helped draft the manuscript. SH, TF, and RN performed the behavioral tests and analyzed the data. TM supervised all aspects of the present study. All authors have read and approved the final manuscript.

## APPROVAL OF THE RESEARCH PROTOCOL BY AN INSTITUTIONAL REVIEWER BOARD

n/a.

## INFORMED CONSENT

n/a.

## REGISTRY AND THE REGISTRATION NO. OF THE STUDY/TRIAL

n/a.

## ANIMAL STUDIES

All experimental procedures were approved by the Institutional Animal Care and Use Committee of Fujita Health University.

## Supporting information

Fig S1‐6

Table S1

Method S1

Supplementary Material

## Data Availability

The data that support the findings of this study are openly available in “Mouse Phenotype Database” at [http://www.mouse‐phenotype.org/], reference number. ^49^
