# Supplementary material for: Decreased nesting behavior, selective increases in locomotor activity in a novel environment, and paradoxically increased open arm exploration in Neurogranin knockout mice
Source: Neuropsychopharmacol Rep. 2020 Dec 3;41(1):111–6. doi: 10.1002/npr2.12150 (PMC8182962; doi:10.1002/npr2.12150)
Supplement: Supplementary file 1 — Fig S1‐6 [file NPR2-41-111-s002.pdf]

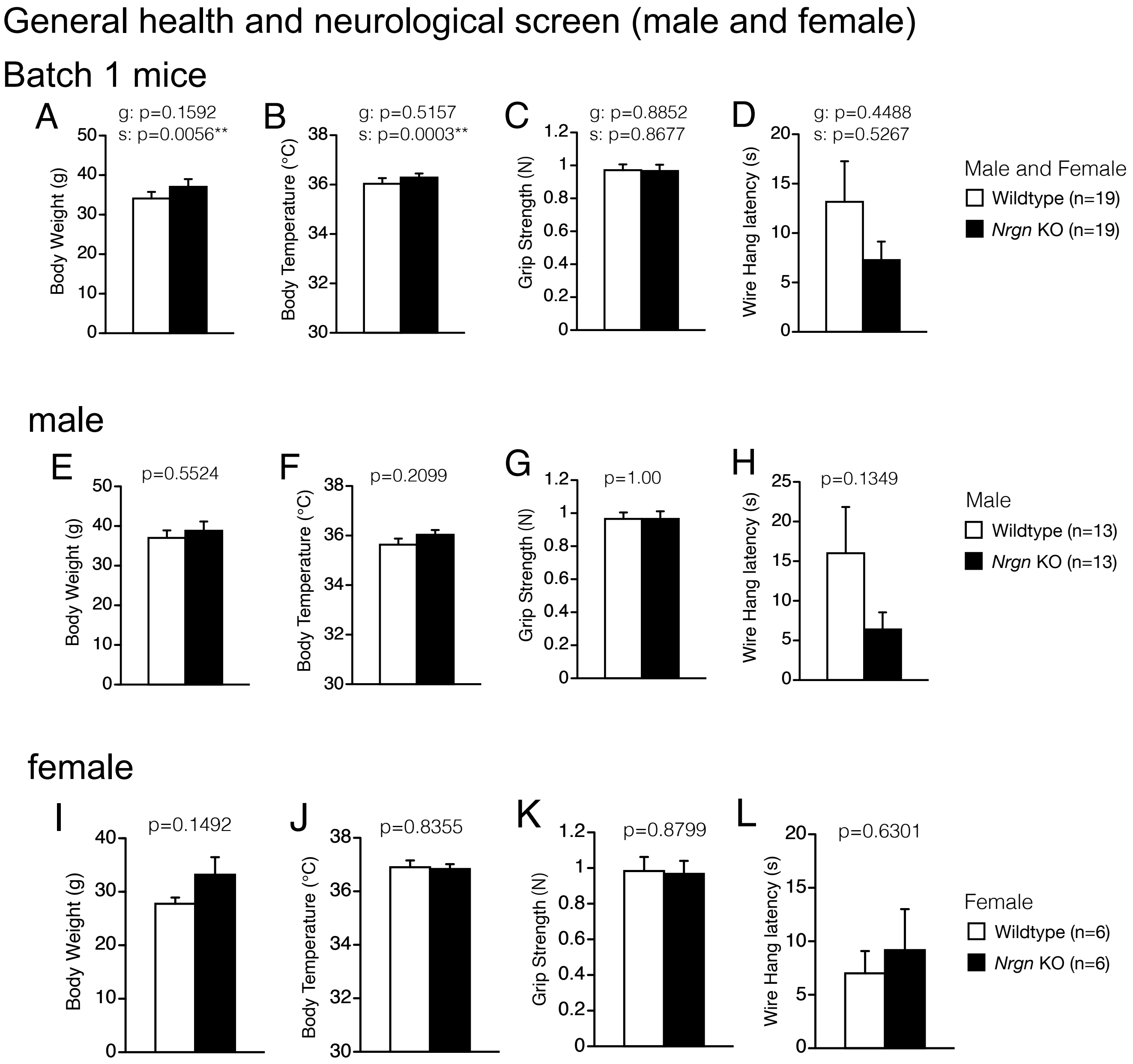

Figure S1. General health and neurological screen, motor learning, and pain sensitivity

(A) Body weight, (B) body temperature, (C) grip strength, and (D) latency to fall in the wire hang test of batch 1 mice. As significant sex effects were detected in some indexes, data for males (E–H) and females (I–L) are also displayed separately. Data are presented as the mean ± SEM. The two-way ANOVA test was used for statistical analysis. Labels of p-values; g: genotype effect; s: sex effect

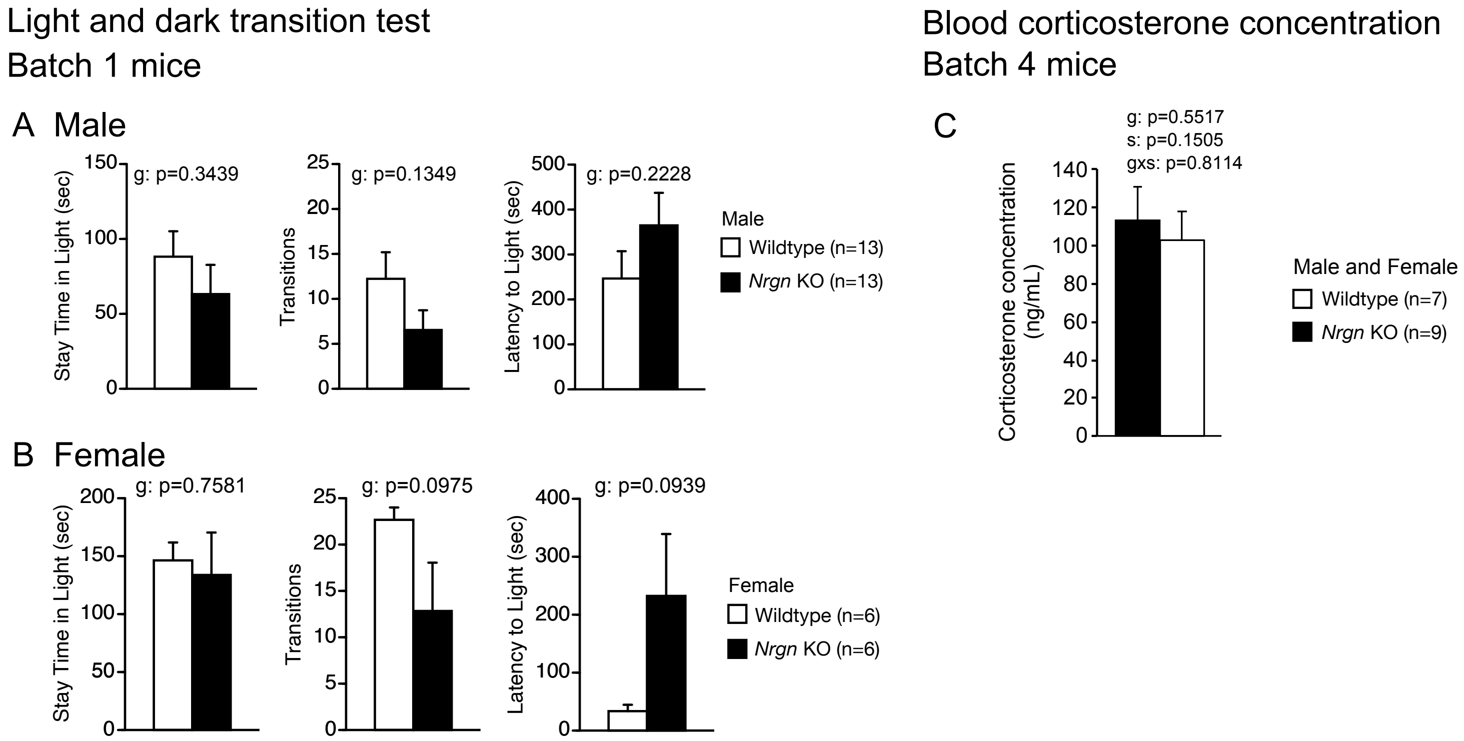

Figure S2. Light/dark transition test and blood corticosterone quantification

(A–B) Light/dark transition test: left panel, time spent in the light compartment; middle panel, number of light/dark transitions; right panel, latency period to enter the light compartment of the batch 1 mice. Due to the significant sex effects in the Light/Dark transition test shown in the main figure, data for males (A) and females (B) are separately displayed. (C) Blood corticosterone concentration in the batch 4 mice after the elevated plus maze. Data are presented as mean ± SEM. The one-way ANOVA (A–B) and two-way ANOVA (C) tests were used for statistical analysis. Labels of p values; g: genotype effect; s: sex effect; g×s : genotype and sex interaction

Open field test

Batch 1 mice

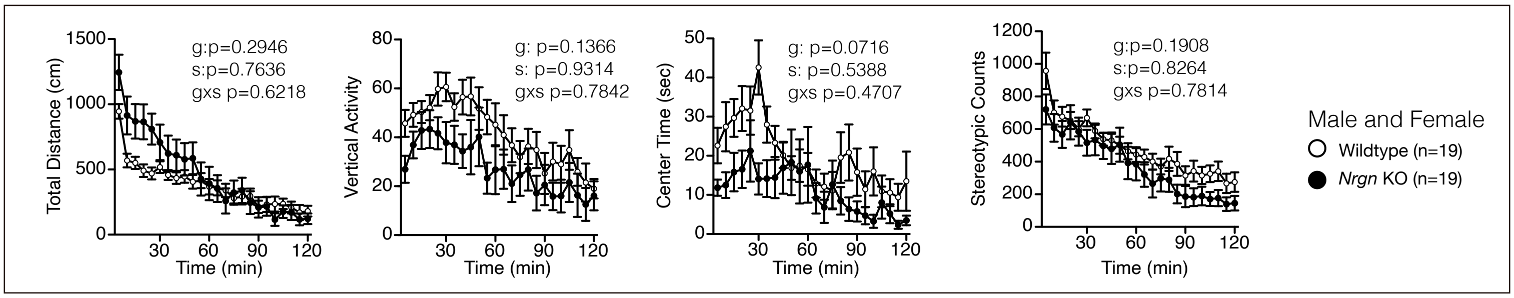

Batch 2 mice

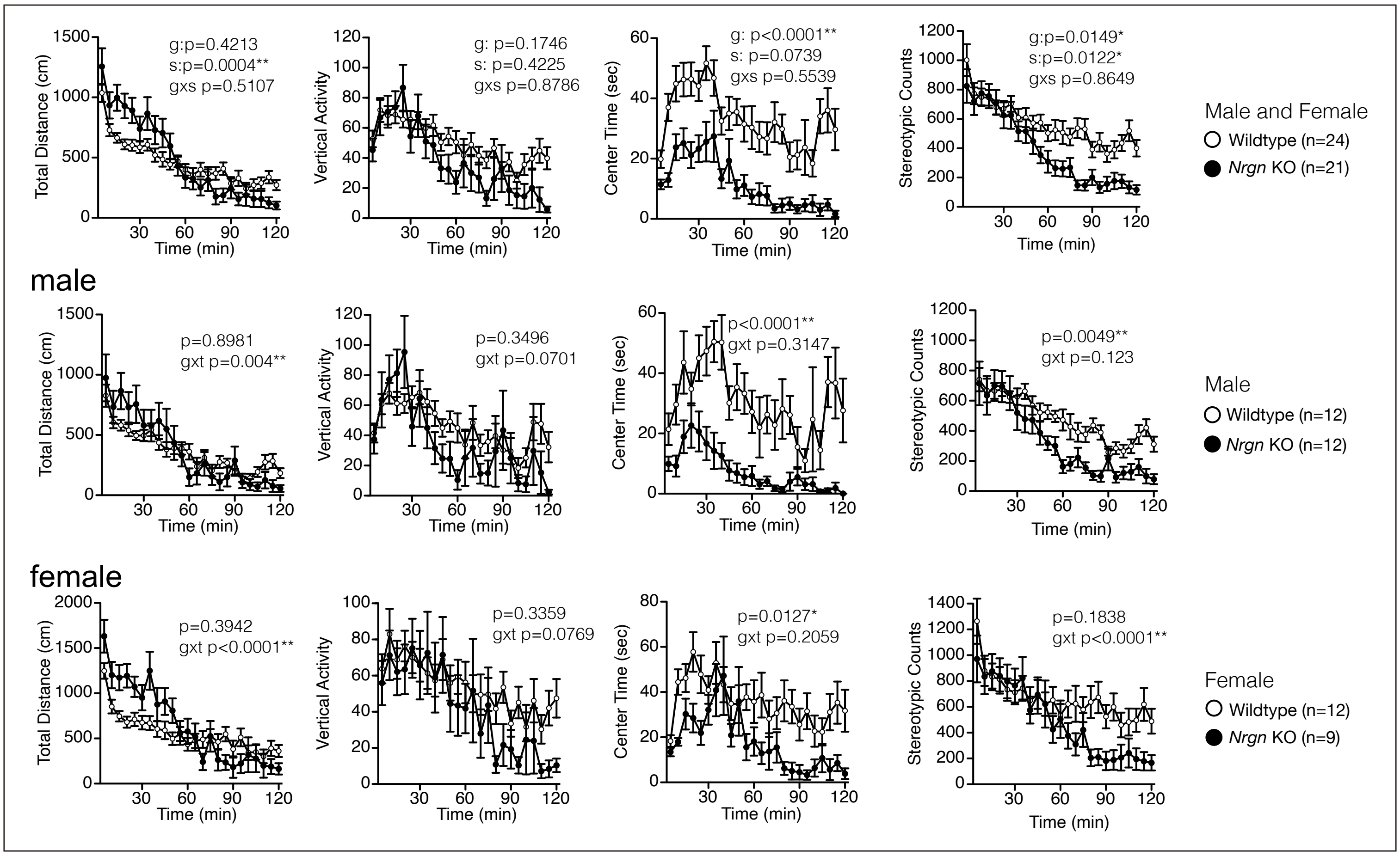

Batch 3 mice

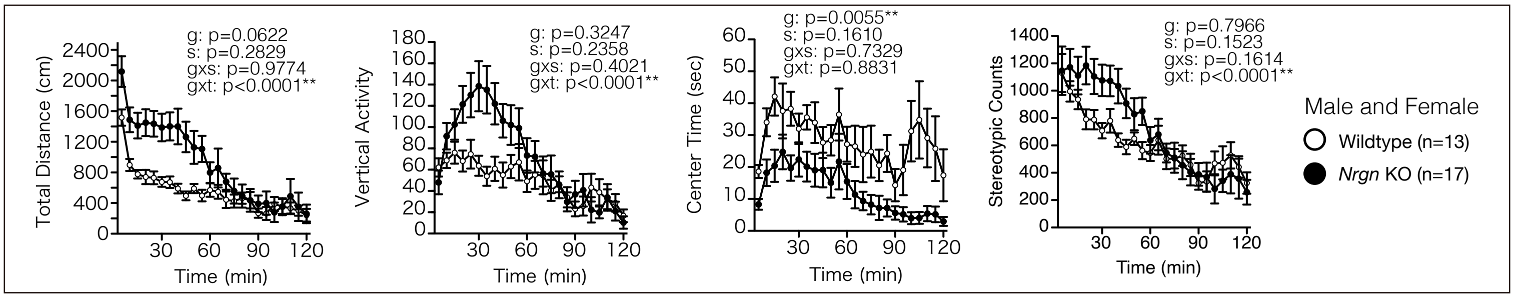

Figure S3. Open field test

The open field test data in the main figure (E) are displayed by sex and mouse batch. (Panels from left to right) total distance traveled, vertical activity, time spent in the center area, and stereotypic behavior counts are represented. Data are presented as mean ± SEM. The two-way or three-way repeated-measures ANOVA was used to analyze the data. Labels of p values; g: genotype effect; s: sex effect; t: time effect; g×s: genotype and sex interaction; g×t: genotype and time interaction

Social interaction test

Batch 1 mice

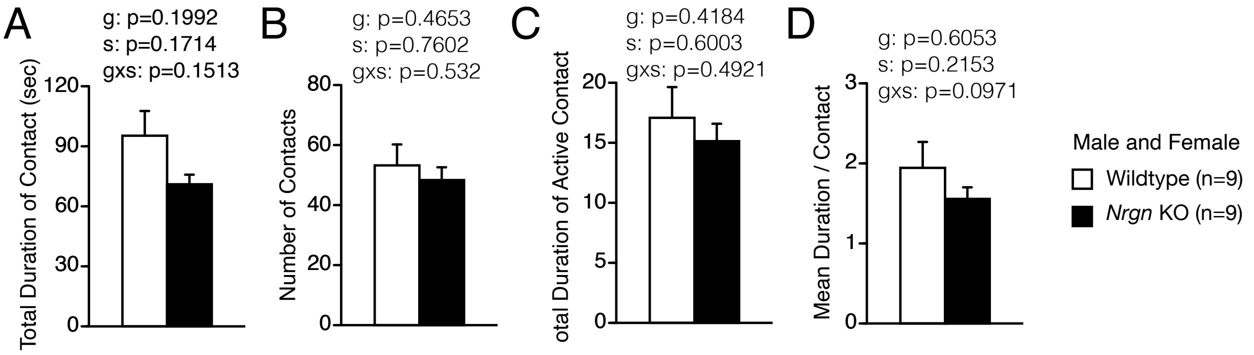

Crawley's sociability and social preference test

Batches 1 and 3 mice

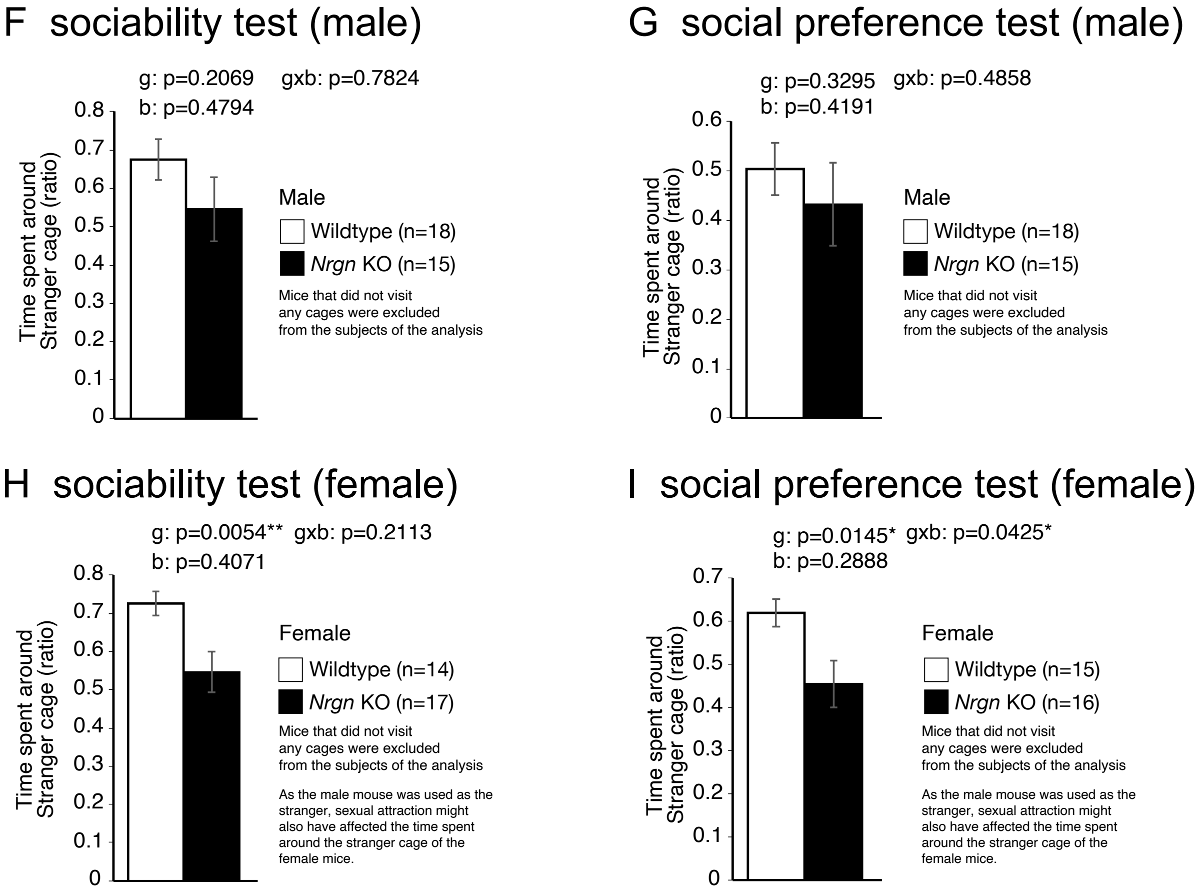

Figure S4. Social interaction tests

(A–D) Social interaction test in novel environments: (A) total duration of contacts, (B) number of contacts, (C) Total duration of active contacts, and (D) mean duration per contact. (F–I) Crawley's sociability and social novelty preference test: ratio of the time spent around the stranger 1 cage in the sociability test (F, male; H, female), ratio of the time spent around the stranger 2 cage in the social novelty preference test (G, male; I, female). As the male mouse was used as the stranger, sexual attraction might also have affected the time spent around the stranger cage of the female mice (H and I). Data are presented as mean ± SEM. Two-way or three-way ANOVA was used to analyze the data. Labels of p values; g: genotype effect; s: sex effect; b: mouse batch effect; g×s: genotype and sex interaction; g×b: genotype and mouse batch interaction.

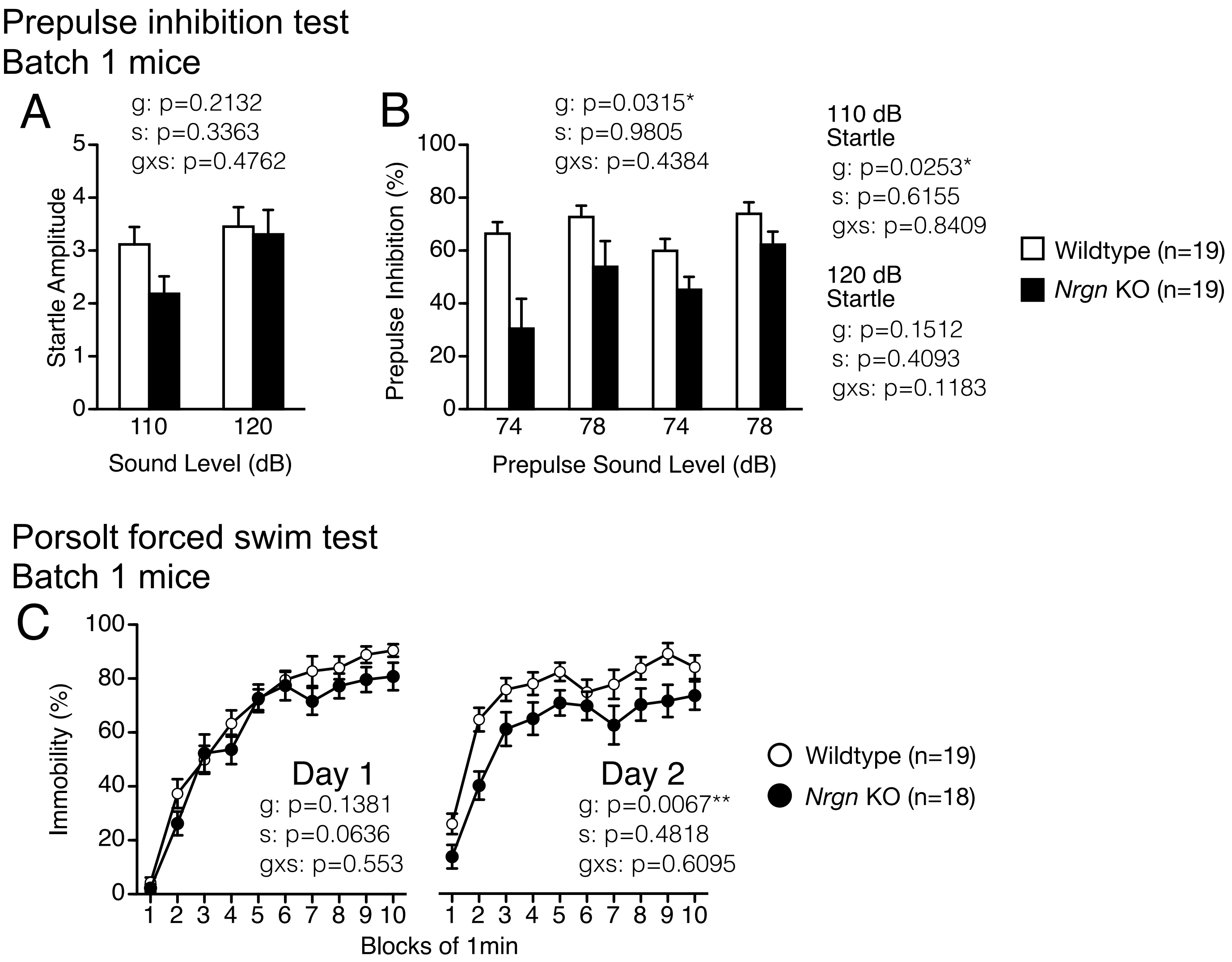

Figure S5. Prepulse inhibition test and Porsolt forced swim test

(A–B) prepulse inhibition test. Startle amplitude (A) and percent of prepulse inhibition (B) were assessed. Data are presented as mean ± SEM. The *P*-values indicate genotype effects in the two-way repeated-measures ANOVA test that was separately performed in experiments with different startle sound levels. (C) Percentage of immobility time on day 1 and day 2 in a Porsolt forced swimming test. Data are presented as mean ± SEM. The *P*-values in (C) indicate genotype effects in the repeated measures ANOVA test. Labels of p values; g: genotype effect, s: sex effect, g×s: genotype and sex interaction.

T-maze spontaneous alternation test

Batches 1 and 3 mice

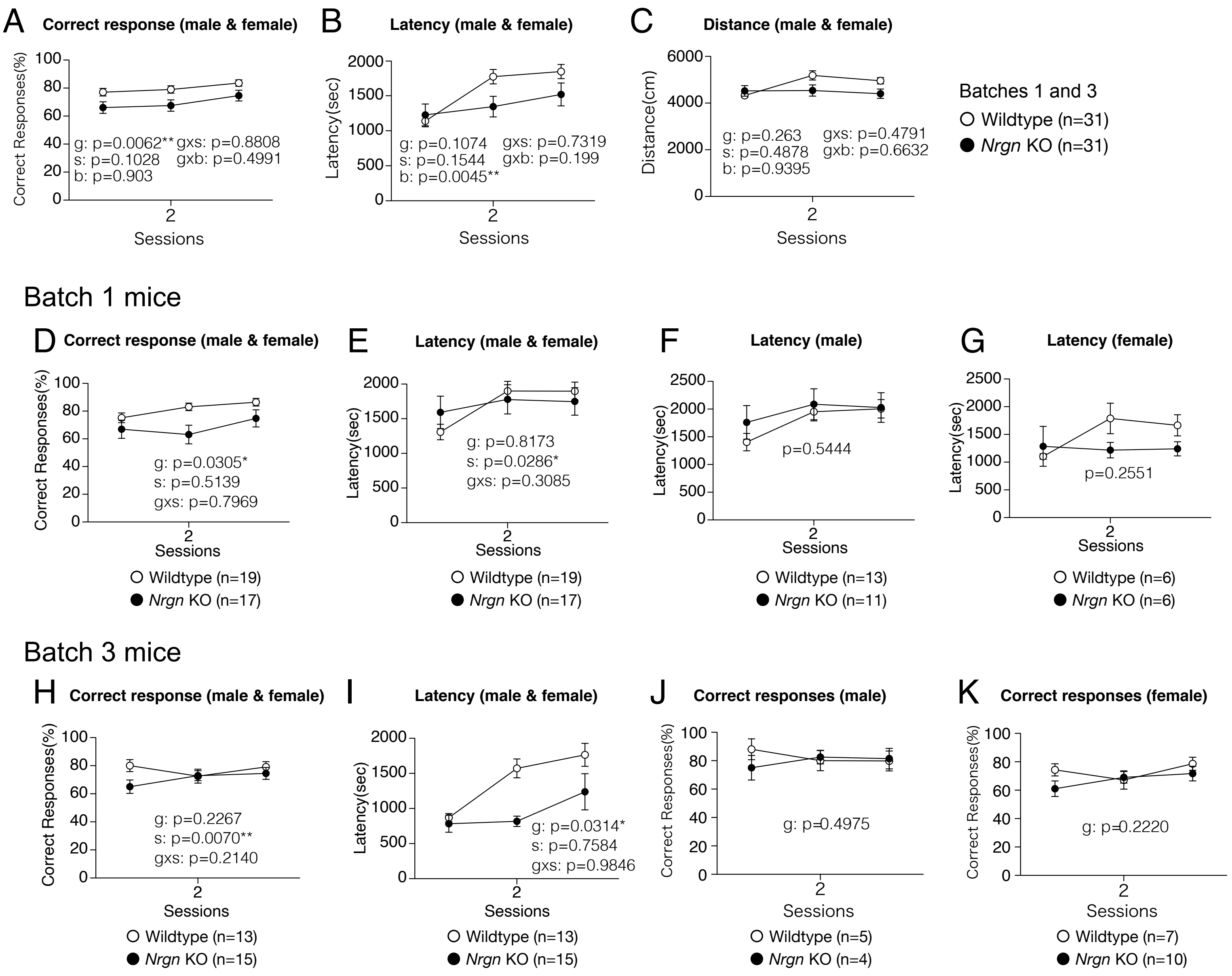

Figure S6. T-maze spontaneous alternation test

(A–C) T-maze spontaneous alternation test of batches 1 and 3 mice. (A) percentage of correct responses, (B) latency (sec), (C) distance traveled (cm). The data were analyzed using the three-way repeated measures ANOVA test. (D–G) T-maze spontaneous alternation test of batch 1 mice. (D) percentage of correct responses, (E) latency (sec), (F) latency of male (sec), (G) latency of female (sec). (H–K) T-maze spontaneous alternation test of batch 3 mice. (H) percentage of correct responses, (I) latency (sec), (J) percentage of correct responses of male, (K) percentage of correct responses of female. The data in (D–K) were analyzed using the repeated measures ANOVA test. Data are presented as mean ± SEM. Labels of p values; g: genotype effect; s: sex effect; b: mouse batch effect; g×s: genotype and sex interaction; g×b: genotype and mouse batch interaction.
