# Supplementary material for: Decreased nesting behavior, selective increases in locomotor activity in a novel environment, and paradoxically increased open arm exploration in Neurogranin knockout mice
Source: Neuropsychopharmacol Rep. 2020 Dec 3;41(1):111–6. doi: 10.1002/npr2.12150 (PMC8182962; doi:10.1002/npr2.12150)
Supplement: Supplementary file 3 — Method S1 [file NPR2-41-111-s004.pdf]

SUPPLEMENTARY METHODS

Animals and experimental design

Nrgn KO mice were generated as previously described (1). The mice were backcrossed to the C57BL/6J mice (Charles river, MA, USA), for at least ten generations, which is also expected to minimize genetic drift. Wild type (WT) and *Nrgn* KO mice of the 1st and 2nd batches were generated by crossing male and female heterozygotes of *Nrgn* mice. The 3rd batch mice were generated by *in vitro* fertilization. Experimental schedules and the age of the mice are indicated in the supplementary table. Mice were group-housed (2–4 mice per cage) in a room with a 12-h light/dark cycle (lights on at 7:00.) with access to food and water ad libitum. The room temperature was kept at 23±2°C. Behavioral testing was performed between 9:00 and 19:00. After the tests, all the testing apparatus were cleaned with diluted hypochlorous solution or 70 % ethanol to prevent a bias due to olfactory cues. All behavioral testing procedures were approved by the Animal Research Committee of Fujita Health University.

Behavioral analysis

Unless otherwise noted, most of the behavioral tests were performed as previously described (2–4).

Neurological Screen and Neuromuscular Strength Test

The righting, whisker twitch, and ear twitch reflexes were evaluated. Physical features, including the presence of whiskers or bald hair patches, were also recorded. A grip strength meter (O'HARA & Co., Tokyo, Japan) was used to assess forelimb grip strength. The peak force applied by the forelimbs of the mouse was recorded in Newtons (N). Each mouse was tested three times, and the greatest value measured was used for data analysis. In the wire hang test, the mouse was placed on a wire mesh that was then slowly inverted, so that the mouse gripped the wire in order not to fall off. Latency to fall was recorded, with a 60 s cut-off time.

Nest-building test

To test the individual nest building behavior, mice were housed individually in cages containing paper chip bedding and one square of pressed cotton, "Nestlets" (Ancare, Bellmore, NY). The manipulation of the Nestlet and the constitution of the built nest were assessed according to a five-point scale as described previously (5)

Light/Dark Transition Test

A light/dark transition test was conducted as previously described (6). The apparatus consisted of a cage with a white floor divided into two sections of equal size (20 × 20 × 25 cm) by a partition with a door (O'HARA & Co., Tokyo, Japan). One chamber was brightly illuminated (390 ± 20 lux), whereas the other chamber was dark (< 2 lux). Mice were placed into the dark chamber and allowed to move freely between the two chambers with the door open for 10min. The total number of transitions, latency to first enter the lit chamber, distance traveled, and time spent in each chamber were recorded by Image LD software (see Section, "Data Analysis").

Open field test

Locomotor activity was measured using an open field test. Each mouse was placed in one corner of the open field apparatus (40 × 40 × 30 cm; Accuscan Instruments, Columbus, OH). Total distance traveled (cm), vertical activity, time spent in the center, the beam-break counts for stereotyped behaviors, and the number of fecal boli were recorded. Data were collected for 120 min.

Elevated plus maze test

The elevated plus maze apparatus consisted of two open arms (25 × 5 cm) and two enclosed arms of the same size, with transparent walls 15 cm high (7). The arms and central square were made of white plastic plates and were elevated 50 cm above the floor. To minimize the likelihood of animals falling from the apparatus, 3 mm-high plexiglass ledges were provided for the open arms. Arms of the same type were arranged on opposite sides. Each mouse was placed in the central square of the maze (5 × 5 cm) facing one of the closed arms. Behavior was recorded during a 10 min test period. For data analysis, the following four measures were employed: the percentage of entries into open arms, the percentage of stay time on the open arms, the number of total entries, and the total distance traveled (cm). Data acquisition and analysis were performed automatically, using an ImageJ-based original program (Image EP: see "Image analysis")

Hot plate test

A hot plate test was used to evaluate sensitivity to a painful stimulus. Mice were placed on a hot plate (Columbus Instruments, Columbus, Ohio) at 55.0 ± 0.3 °C, and latency to the first paw response was recorded. A paw response was a foot shake, or a paw lick, or lifting both forepaws simultaneously.

Social interaction test in a novel environment

The social interaction test in a novel environment was performed with 32-40 weeks old mice. Two mice of identical genotypes that were previously housed in different cages were placed into a box together (40 × 40 × 30 cm) and allowed to explore freely for 10 min. Analysis was performed automatically using Image SI software (see "Image analysis"). The total duration of contact, number of contacts, number of active contacts, mean duration per contact, and total distance traveled were measured.

Rotarod Test

Motor coordination and balance were tested using the rotarod test. This test, which uses an accelerating rotarod (UGO Basile, Comerio, VA, Italy), was performed by placing mice on rotating drums, and measuring the time each animal was able to maintain its balance on the rod. The speed of the rotarod was accelerated from 4 to 40 rpm over a 5-min period.

Sociability and social novelty preference test

The social testing apparatus consisted of a rectangular, three-chambered box and a lid fitted with a camera (Ohara & Co., Tokyo). Each chamber was 20 × 40 × 46.5 cm in size, and the dividing walls were made from clear plexiglass, with small square openings (5 × 3 cm) allowing access into each chamber. An unfamiliar C57BL/6J male (stranger 1) was placed in one of the side chambers. The location of stranger 1 in the left vs. the right side chamber was systematically alternated between trials. The stranger mouse was enclosed in a small, round wire cage, which allowed nose contact between the bars but prevented fighting. The cage was 11 cm in height, with a bottom diameter of 9 cm, vertical bars 0.5 cm. The subject mouse was first placed in the middle chamber and allowed to explore the entire social test box for a 10 min session to quantify social approach for the first stranger. After the first 10 min session, a second unfamiliar mouse, also enclosed in an identical small wire cage, was placed in the chamber that had been empty during the first 10 min session. The mouse subjected to the test thus had a choice between the first, already-investigated unfamiliar mouse (stranger 1), and the novel unfamiliar mouse (stranger 2). The amount of time spent around each cage during the second 10-minutes was measured. Ratio of the time spent around stranger cage, in total time spent around both cages, compared using a two-way ANOVA. Data acquisition and analysis were performed

automatically using an ImageJ based original program (Image CSI: see "Image analysis").

Startle response/prepulse inhibition test

A startle reflex measurement system was used (O'Hara & Co., Tokyo). A test session began by placing a mouse in a plexiglass cylinder, where it was left undisturbed for 10 min. A 40 ms duration of white noise was used as the startle stimulus for all trial types. The background noise level in each chamber was 70 dB. A test session consisted of six trial types (i.e., two types for "startle-stimulus-only" trials and four types for prepulse inhibition (PPI) trials). The intensity of the startle stimulus was 110 or 120 dB. The prepulse sound was presented 100 ms before the startle stimulus, and its intensity was 74 or 78 dB. The peak startle amplitude was used as the dependent variable. Six blocks of the six trial types were presented in pseudo-random order such that each trial type was presented once within a block. The average inter-trial interval was 15 s (range: 10–20 s).

Porsolt forced swim test

The apparatus consisted of four plexiglass cylinders (21.5 cm height × 11 cm diameter). The cylinders were filled with water (23±2°C.), up to a height of 7.5 cm. Mice were placed in the cylinders, and immobility and distance traveled were recorded over a 10 min test period. Data acquisition and analysis were performed automatically using an ImageJ based original program (Image TS: see "Image analysis").

T-maze spontaneous alternation task

The spontaneous alternation task was conducted using an automatic T-maze apparatus (O'HARA & Co.) as previously described (8). Mice were subjected to a spontaneous alternation protocol for 3 sessions, with at least 1 day (2 days maximum) of session-to-session intervals. One session consists of 10 trials with a 50-minute cutoff time. Each trial had first and second runs. On the first run, the mouse was forced to choose one of the arms of the T, and the mouse could return to the starting compartment. The mouse was then given a 3-sec delay followed by a free choice between both T arms. The percentage of trials in which mice entered the arm opposite to their forced-choice run during the free choice run was calculated. Total time spent and total distance traveled during the sessions were also recorded. Data acquisition, control of sliding doors, and data analysis were performed by ImageTM software (see Section, "Data Analysis").

Tail Suspension Test

The tail suspension test was performed for a 10-min test session. Mice were suspended approximately 30 cm above the floor of a white plastic chamber (32 × 49 × 44 cm) (O'HARA & Co.), and the behavior was recorded over a 10-min test period. Immobility (%) was judged by the application program. Data acquisition and analysis were performed automatically using ImageTS software (see Section "Data analysis").

Locomotor activity monitoring in the home cage

A system that automatically analyzes the locomotor activity of mice in their home cage was used. The system contained a home cage and a filtered cage top. Each mouse was individually housed in each home cage, and their locomotor activity was monitored for 1 week. Distance travelled was measured automatically using Image HC software (see "Image analysis").

Image analysis

The applications used for the behavioral studies (Image LD, Image EP, Image SI, Image TS, Image TM, Image CSI and Image HC) were based on the public domain ImageJ program

(http://rsb.info.nih.gov/ij/) and were modified for each test by the authors (available through O'Hara & Co., Tokyo, Japan).

Statistical analysis

Statistical analysis was conducted using StatView (SAS Institute, Cary, NC). Data were analyzed by one-way ANOVA, two-way ANOVA or two-way repeated-measures ANOVA unless noted otherwise. Values in Tables and graphs are expressed as the mean ± standard error of the mean (SEM).

REFERENCES FOR SUPPLEMENTARY METHODS

1. Pak JH, Huang FL, Li J, Balschun D, Reymann KG, Chiang C, et al. Involvement of neurogranin in the modulation of calcium/calmodulin-dependent protein kinase II, synaptic plasticity, and spatial learning: A study with knockout mice. PNAS. 2000 Oct 10;97(21):11232–7.

2. Yamasaki N, Maekawa M, Kobayashi K, Kajii Y, Maeda J, Soma M, et al. Alpha-CaMKII deficiency causes immature dentate gyrus, a novel candidate endophenotype of psychiatric disorders. Molecular Brain. 2008 Sep 10;1(1):6.

3. Yoshioka N, Miyata S, Tamada A, Watanabe Y, Kawasaki A, Kitagawa H, et al. Abnormalities in perineuronal nets and behavior in mice lacking CSGalNAcT1, a key enzyme in chondroitin sulfate synthesis. Molecular Brain [Internet]. 2017 Dec [cited 2019 Mar 25];10(1). Available from: http://molecularbrain.biomedcentral.com/articles/10.1186/s13041-017-0328-5

4. Katano T, Takao K, Abe M, Yamazaki M, Watanabe M, Miyakawa T, et al. Distribution of Caskin1 protein and phenotypic characterization of its knockout mice using a comprehensive behavioral test battery. Molecular Brain [Internet]. 2018 Dec [cited 2019 Mar 25];11(1). Available from: https://molecularbrain.biomedcentral.com/articles/10.1186/s13041-018-0407-2

5. Deacon RMJ. Assessing nest building in mice. Nat Protoc. 2006;1(3):1117–9.

6. Takao K, Miyakawa T. Light/dark transition test for mice. J Vis Exp. 2006 Dec;(1):104.

7. Komada M, Takao K, Miyakawa T. Elevated plus maze for mice. J Vis Exp [Internet]. 2008 [cited 2011 Sep 28];(22). Available from: http://www.ncbi.nlm.nih.gov/pubmed/19229173

8. Shoji H, Hagihara H, Takao K, Hattori S, Miyakawa T. T-maze Forced Alternation and Left-right Discrimination Tasks for Assessing Working and Reference Memory in Mice. J Vis Exp [Internet]. 2012 [cited 2012 Apr 5];(60). Available from: http://www.ncbi.nlm.nih.gov/pubmed/22395674
