# Supplementary material for: Decreased nesting behavior, selective increases in locomotor activity in a novel environment, and paradoxically increased open arm exploration in Neurogranin knockout mice
Source: Neuropsychopharmacol Rep. 2020 Dec 3;41(1):111–6. doi: 10.1002/npr2.12150 (PMC8182962; doi:10.1002/npr2.12150)
Supplement: Supplementary file 4 — Supplementary Material [file NPR2-41-111-s001.docx]

**SUPPLEMENTARY MATERIALS**

All the data that are not shown in the main figure, methods, and summary of all the results, including the significance of the genotype effect, sex effect, mouse batch effect, and the interactions between the effects, are available as supplementary materials.

**SUPPORTING INFORMATION**

**Supplementary figures:** The figures that are not shown in the main figure are available in the supplementary figures. For data where a significant sex effect was observed, the male and female data are shown separately.

**Supplementary table:** Summary of all the results and ages of the mice in the present study are available in the supplementary table.

**Supplementary methods:** All the methods and the references for the methods are available in the supplementary methods.
